# Comparative Oncology: New Insights into an Ancient Disease

**DOI:** 10.1016/j.isci.2020.101373

**Published:** 2020-07-16

**Authors:** Amy M. Boddy, Tara M. Harrison, Lisa M. Abegglen

**Affiliations:** 1 Department of Anthropology, University of California Santa Barbara, Santa Barbara, CA, USA; 2 Department of Clinical Sciences, College of Veterinary Medicine, North Carolina State University, Raleigh, NC, USA; 3 Department of Pediatrics, University of Utah, Salt Lake City, UT, USA; 4 Huntsman Cancer Institute, University of Utah, Salt Lake City, UT, USA

**Keywords:** Evolutionary Biology, Cancer

## Abstract

Cancer has deep evolutionary roots and is an important source of selective pressure in organismal evolution. Yet, we find a great deal of variation in cancer vulnerabilities across the tree of life. Comparative oncology offers insights into why some species vary in their susceptibility to cancer and the mechanisms responsible for the diversity of cancer defenses. Here we provide an overview for why cancer persists across the tree of life. We then summarize current data on cancer in mammals, reptiles, and birds in comparison with commonly reported human cancers. We report on both novel and shared mechanisms of cancer protection in animals. Cross-discipline collaborations, including zoological and aquarium institutions, wildlife and evolutionary biologists, veterinarians, medical doctors, cancer biologists, and oncologists, will be essential for progress in the field of comparative oncology. Improving medical treatment of humans and animals with cancer is the ultimate promise of comparative oncology.

## Graphical Abstract

**Figure:**
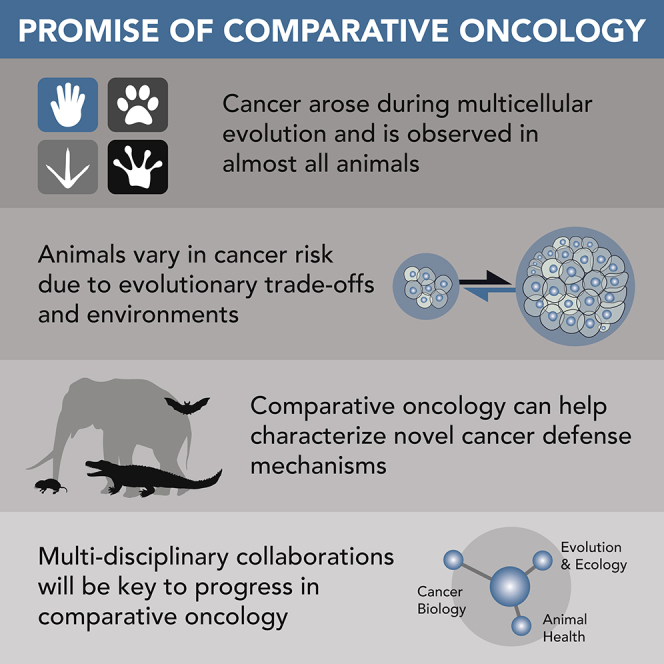

## Teaser

Evolutionary Biology; Cancers

## Consequences of (Multicellular) Cooperation

**Figure 1:**
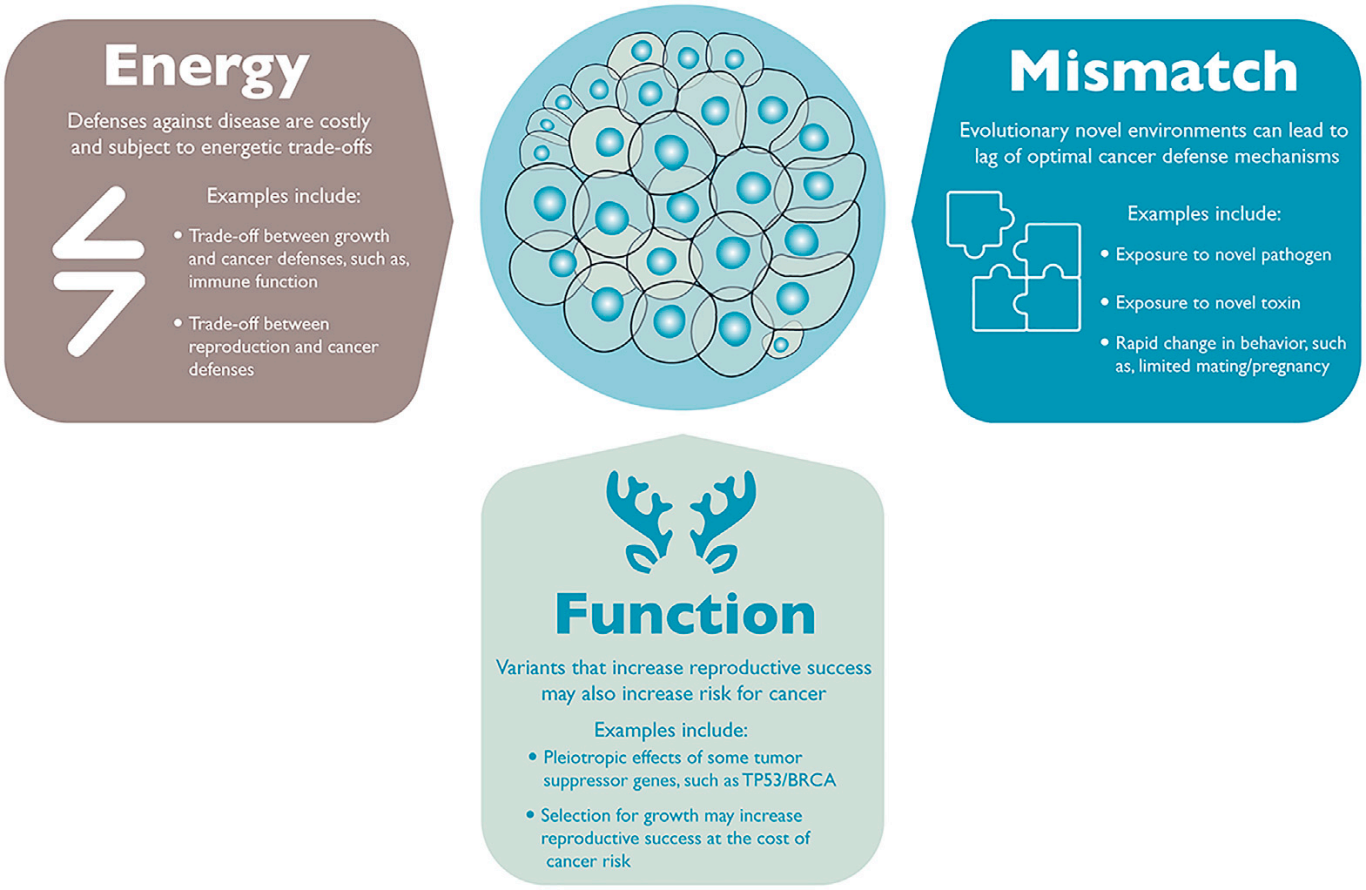
Why Evolution Has Not Solved the Problem of Cancer Here we illustrate three evolutionary perspectives for why we observe cancer across the tree of life. We include examples for each evolutionary hypothesis. These hypotheses are not mutually exclusive and include (clockwise) energetic life history trade-offs, evolutionary mismatch with the environment, and functional trade-offs, such as antagonistic pleiotropy.

Cancer is a consequence of multicellular evolution (Aktipis et al., 2015; Nunney and Muir, 2015), and as such, cancer has imposed persistent selective pressure on multicellular organisms for the past three billion years. If cancer is evolutionarily ancient, why haven't all organisms solved the problem of cancer? Indeed, many mechanisms of cancer defense evolved across the tree of life. For example, animals have physical defenses, including tissue architecture, and molecular defenses, such as multiple cell-cycle checkpoints, redundant cell cycle pathways, and DNA damage response (including DNA repair and apoptosis) (DeGregori, 2011; Harris et al., 2017). Furthermore, the emergence of tumor suppressor genes can be traced with the emergence of multicellular metazoans (Domazet-Lošo and Tautz, 2010). Yet, beyond these cancer defenses, neoplasia is still a problem for many animals (Aktipis et al., 2015; Albuquerque et al., 2018; Lingeman and Garner, 1969; Madsen et al., 2017; McAloose and Newton, 2009), including humans. Here we highlight three (not mutually exclusive) evolutionary perspectives for why cancer is a problem for many animals and why vulnerabilities to cancer vary across the tree of life (Figure 1).

### Trade-Offs: Life History Theory and Antagonistic Pleiotropy

Species may be more vulnerable to cancer owing to trade-offs and constraints (Jacqueline et al., 2016; Rozhok and DeGregori, 2016). Here we distinguish between energetic versus function trade-offs (Maklakov and Chapman, 2019). Although they are not mutually exclusive, we propose fundamental differences between these types of trade-offs, which we outline below as: (1) life history trade-offs (i.e., energetic trade-offs) and (2) antagonistic pleiotropy (i.e., function trade-offs).

#### Energetic Trade-Offs: Life History Theory and Cancer Vulnerabilities

A life history framework can help explain the large pattern of variation in cancer vulnerabilities across animals (Boddy et al., 2015; Kokko and Hochberg, 2015). According to life history theory, animals maximize fitness through differing rates of survival, growth/maintenance, and reproduction (Stearns, 1989). Owing to finite resources, these life history traits are subject to trade-offs, which we define as energetic trade-offs. For example, an organism that allocates resources toward growth, typically does so at the expense of reproductive output (Stearns, 1989). These life history traits fall on a “fast-slow life history continuum,” ranging from fast life history traits, such as quick growth, short lifespan, and high reproductive output, to slow life history traits, such as slow growth, long lifespan, and low reproductive output. Estimates suggest a large percentage (71%) of the variation in life history strategies are associated with this fast-slow continuum (Healy et al., 2019). Importantly, this life history perspective provides comparative oncology with a theoretical framework to generate specific predictions. We can predict animals that maximize growth and somatic maintenance over reproduction (i.e., slow life history animal, including elephants and whales) will develop less cancer than animals that maximize reproduction over lifespan (i.e., fast life history animal, including mice and hedgehogs). Cancer suppression is a major component of somatic maintenance, which requires cell cycle control, DNA repair, and immune function (Boddy et al., 2015). The idea that large, long-lived animals develop less cancer was first observed by cancer biologists in the 1960s, and this observation became known as “Peto's Paradox” (Peto, 2015; Peto et al., 1975). If each dividing cell has equal risk for accumulating cancer-inducing mutations, then species with more cells (owing to larger body mass) or longer lifespan (more time to accumulate mutations) should have a higher probability of developing mutations that lead to cancer (Caulin et al., 2015; Nunney and Muir, 2015). Peto's Paradox proposed that bigger, longer-lived animals do not develop more cancer than small-bodied, short-lived species, despite this increased risk for developing mutations. Current comparative oncology data support Peto's Paradox and report no relationship between body mass, lifespan, and cancer risk (Abegglen et al., 2015; Boddy et al., 2020), possibly owing to evolution of additional mechanisms of tumor suppression in large, long-lived species (Abegglen et al., 2015; Sulak et al., 2016; Tollis et al., 2019).

Although the above framework focuses on variation of cancer across species, energetic trade-offs may also help explain within-species variation (Boddy et al., 2015; Hochberg and Noble, 2017). These trade-offs in somatic maintenance (and cancer defenses) depend on environmental availability of resources and extrinsic mortality conditions. Evidence suggests that a robust immune system is costly, and there is a potential trade-off between immune function and growth. For example, a meta-analysis of immune function and growth in commercial poultry found selection for body mass compromised immune function in experimental conditions (Most et al., 2011). Additionally, external conditions, including food availability, disease, predators, and reproductive competition, may influence somatic maintenance strategies (Boddy et al., 2015; Rozhok and DeGregori, 2016), especially when external conditions drastically change (see section on Mismatch).

#### Function Trade-Offs: Antagonistic Pleiotropy

Antagonistic pleiotropy, which we describe as a functional trade-off, is another important concept in evolutionary biology, especially in regards to aging. This concept requires two assumptions to be met and, although it is sometimes used interchangeably with energetic trade-offs, antagonistic pleiotropy is quite distinct from the life history trade-offs described in the previous section. One of the first assumptions of antagonistic pleiotropy is the evidence of pleiotropy, meaning a gene or allele controls more than one phenotype. Second, the phenotypes must influence the fitness of the organism in opposing directions (i.e., beneficial and detrimental) making the pleiotropy antagonistic. If a phenotype provides benefits on reproductive success early in life, the allele could be selected for even if the allele is responsible for a deleterious trait that occurs later in life (Williams, 1957). In other words, disease-causing alleles can persist in a population owing to the early life positive benefits. Effects of antagonistic pleiotropy are ubiquitous across the tree of life (Austad and Hoffman, 2018) and provide a mechanism for the positive selection and persistence of cancer-promoting genes in populations (Campisi, 2013; Crespi and Summers, 2006). Some examples of antagonistic pleiotropy in cancer biology include tumor suppressor variants that may also functionally contribute to increased fertility, such as *BRCA* 1/2 (Smith et al., 2013), *TP53* (Kang et al., 2009), and *KISS* (Mumtaz et al., 2017). Each of these genes may have pleiotropic effects that increase an organism's vulnerability to cancer.

Bigger organisms are generally less susceptible to predation and have increased opportunities for reproductive success over time. Yet, as discussed above, a large body mass requires more cells and more cell divisions, which increase the chance of deleterious mutations. Genes important for growth, such as hormones and growth factors, may be subject to pleiotropic effects. Higher circulating levels of growth factors benefit chances of reproductive success but could increase the chance of cancer later in life. The most well-known example of this pleiotropic effect is from a species of platyfish, *Xiphophorus*. A subset of these fish harbor a polymorphism in a growth factor, *Xmrk* (Schartl et al., 1999). High expression of this *Xmrk* variant can lead to the development of melanoma. However, male platyfish with the *Xmrk* variant (and subsequent melanoma) are significantly larger, and females preferentially mate with the largest males (Fernandez and Bowser, 2010; Fernandez and Morris, 2008). The *Xmrk* variant in platyfish provides an example of how an allele that increases cancer risk may persist in a population owing to early life benefits on reproductive success.

### Evolutionary Mismatch Hypothesis

Cancer defenses evolved in response to a species environment, including features of the environment that may pose a threat to DNA integrity, such as sunlight, toxins, and viruses. Deviations from a species natural habitat can contribute to cancer risk (Hochberg and Noble, 2017; Pesavento et al., 2018; Sepp et al., 2019). This concept is called evolutionary mismatch and corresponds to when the environment changes faster than the population can adapt (Gluckman et al., 2019). Although often characterized for its deleterious effects, the context of a rapidly changing environment does not always produce deleterious health effects, including cancer risk, for the local population. Some species are able to rapidly adapt, and not all environmental changes lead to worse health outcomes. However, for the purpose of this review article, we will highlight a few areas of environmental mismatch that may influence the risk of cancer in human and wildlife populations. Commonly cited examples of how environmental changes may increase cancer risk in human populations include skin cancer (Jablonski and Chaplin, 2010), breast cancer (Aktipis et al., 2014), and childhood acute lymphoblastic leukemia (Greaves, 2018).

Wildlife populations are also vulnerable to evolutionary mismatch. Currently, wildlife populations face global climate change, deforestation, reduced population sizes, and novel exposures to pollution and pathogens. Accordingly, we are likely to observe more species rapidly shifting into environmental mismatch. Generation time, the average time required for individuals in a population to reach sexual maturity and reproduce, dramatically impacts how quickly species can respond to environmental changes (Staerk et al., 2019). Generation time varies greatly between species. Populations with short generation times may rapidly adapt, whereas species with long generation times may be the most vulnerable to environmental mismatch. Although not all environmental perturbations influence oncogenic risk in wildlife, there are many examples of wildlife developing tumors and cancer linked to environmentally induced toxins and pollution, including metastatic carcinoma of urogenital origin in the California sea lion (Browning et al., 2015), gastrointestinal epithelial cancers in a population of beluga whales (Martineau et al., 2002), and fibropapillomas in green sea turtles (Foley et al., 2005). We are unsure how global changes in the environment will affect the development of cancer in animals, but anthropogenic activities that increase wildlife exposure to pollution can likely contribute to the development of neoplasia in wildlife species (McAloose and Newton, 2009).

In addition to novel pathogens and toxin exposure, reduction in population size (i.e., population bottlenecks from novel disease, deforestation) leads to reduced genetic diversity that can affect disease risk. A few case studies suggest that reduced genetic diversity may also contribute to cancer vulnerabilities (Ujvari et al., 2018). Of special note is the link between population decline and cancer in the charismatic Tasmanian devils. Tasmanian devils are vulnerable to transmissible facial tumors, known as Devil Facial Tumor Disease (DFTD). DFTD is transmitted through animals biting and has caused widespread disease in the population (Murchison, 2008; Stammnitz et al., 2018). Tasmanian devils underwent a population bottleneck, resulting in low genomic diversity in genes involved in immune response, including the Major Histocompatibility Complex (MHC) genes (Siddle et al., 2010). This lack of diversity at MHC loci and reduced effectiveness of the immune response may have left the devils more vulnerable to transmissible cancer (Murchison, 2008). Interestingly, Tasmanian devils housed at the San Diego Zoo, which live in a protected environment with no exposure to DFTD, still developed a high prevalence of neoplasia and malignant cancer (>30% of the individuals diagnosed with malignant neoplasia) (Abegglen et al., 2015; Boddy et al., 2020). Although larger datasets are needed to validate these findings, the observations suggest an overall weak defense to combat tumors within Tasmanian devils. Similar links between reduced genomic diversity, including MHC genes, and cancer have been reported in California sea lions (Acevedo-Whitehouse et al., 2003; Browning et al., 2015). Additionally, cancer susceptibility among wildlife poses serious consequences for endangered species and warrants further investigation to the underlying mechanisms responsible. For example, endangered Santa Catalina Island fox population have remarkably high prevalence of ear tumors, which has been directly linked to ear mite-induced inflammation (Vickers et al., 2015), and the endangered population of Mexican wolves are susceptible to squamous cell carcinoma, adenocarcinoma, and fibrosarcoma (Sanchez et al., 2012).

Lastly, we should also note that domesticated species, including dogs, cats, and chickens, accumulate deleterious alleles owing to selective breeding (artificial selection), which is associated with increased risk for developing specific types of neoplasia and malignancy (Johnson and Giles, 2013; Schiffman and Breen, 2015). Increased cancer in domestic species is perhaps most notably observed in purebred dog species. Purebred dogs are bred for appearance and behavior. As a result, they are susceptible to breed-specific cancers such as osteosarcoma in Great Danes and Rottweilers, hemangiosarcoma in German Shepherds, and mast cell tumors and glioblastoma in Boxers (Schiffman and Breen, 2015). Interestingly, although Peto's Paradox exists *across* species (larger animals do not get more cancer than smaller animals), this paradox does not occur *within* certain species, including dogs.

## Summary of Current Comparative Oncology Data

Neoplasia in invertebrates, fish, reptiles, and amphibians were some of the first wildlife cancers documented and studied through the Registry of Tumors in Lower Animals, a neoplasia database first organized from 1965 to 1985 through a collaboration between the National Cancer Institute and the Smithsonian Institution (Harshbarger, 1969). This registry documented cases of neoplasia and their relationship to environmental pollution. Although this registry is no longer active, it expanded the understanding of neoplasia and malignant cancer risk beyond human medicine. Importantly, it documented the potential contribution of humans to cancer risk in animals due to environmental pollution of animal habitats.

In the years since the Registry of Tumors in Lower Animals was active, numerous published case studies have reported neoplasia and malignant cancer in vertebrates. Most of these case studies are from animals housed and managed in various zoological and aquarium institutes, because it is easier to diagnose cancer in animals receiving routine medical care and health examinations. However, a few case reports are from wild animals (reviewed in Madsen et al., 2017), and a limited number of comprehensive surveys of cancer prevalence in wild animals exist (Bowen et al., 2005; Effron et al., 1977; Møller et al., 2017; Wolfe and Spraker, 2007). Collecting accurate neoplasia data in wild animals is challenging. Comprehensive necropsies (known as autopsies in human populations) to detect tumors in a wild animal must occur before the body is consumed by predators or before the tissue degrades in the field. However, the diagnosis and characterization of cancer in wild animals are critical next steps for comparative oncology. Fortunately, many megavertebrates, such as elephants and rhinoceroses, are now closely managed and monitored by wildlife veterinarians, biologists, and researchers, which results in wildlife medical diagnoses, including cancer (Pesavento et al., 2018). These heightened observations and medical examinations may offer an important opportunity to observe and quantify cancer in wild animals.

We currently do not know how living in a protected, semi-natural environment with routine medical care (and potentially less exposure to a diversity of pathogens and predators) may influence cancer rates in zoological and aquarium animals compared with their wild counterparts. As we stated above, environmental conditions can influence a species vulnerability to cancer. Additionally, animals housed in zoos and aquariums tend to live longer than in the wild because of reduced risk of predation. However, in the case of the Tasmanian devil, current zoological and wildlife data suggest these animals are highly susceptible to cancer. Interestingly, the types of cancer they develop differ between the two environments (Abegglen et al., 2015; Boddy et al., 2020; Stammnitz et al., 2018). Whether this pattern applies across other animals is currently unknown but is worthy of further investigation.

Diagnosing neoplasia in zoological settings also poses challenges. Not all animal facilities have the resources and support needed to collect biopsy and necropsy tissues for analysis by board certified veterinarian pathologists for diagnoses. Additionally, institutional data may be biased, because medical examinations and testing are more often performed on charismatic species, such as the large, long-lived mammals, which results in more cancer diagnoses and treatment in these populations compared with less charismatic species. However, most institutional members of the Association of Zoos and Aquariums (AZA) perform necropsies on all species housed in their facilities. Separate analysis of data from institutions that follow routine, standardized necropsy protocols for all species may eliminate biases associated with medical care and necropsies focused on subsets of animals. Collating data from many different zoological institutions, with many different methods for collecting medical records, will continue to be a major challenge for reporting cancer incidence in a broader comparative setting. Studies consisting of data from single facilities lead to small sample sizes with large confidence intervals. However, these data are still valuable and worthy of collection, analysis, and publication.

**Figure 2:**
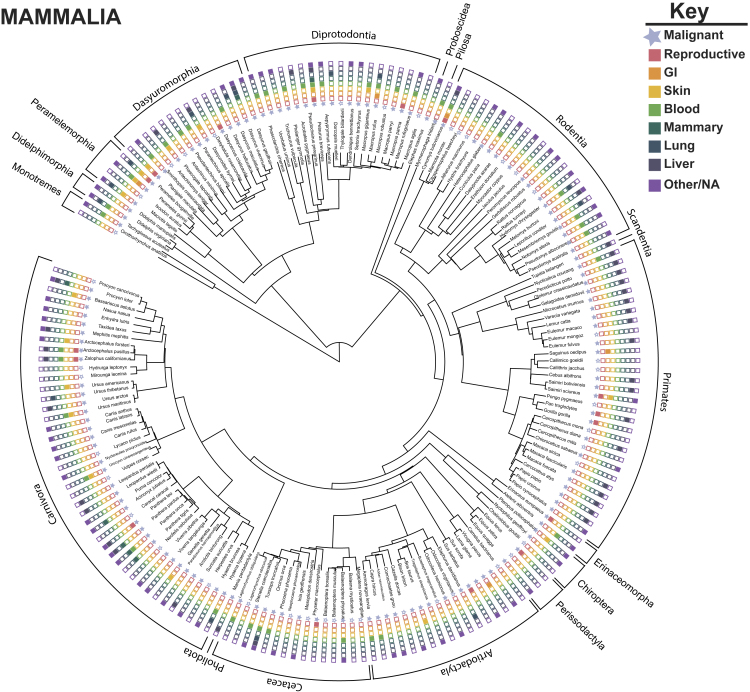
Phylogeny of Reported Cancers and Benign Tumors in Mammalia Illustration of the comprehensive Madsen et al. (2017) literature review of reported neoplasia/tumors and malignancy in mammals. Species represented in the phylogeny had tumors reported. Phylogeny was inferred from VertLife (Upham et al., 2019) created using the Interactive Tree of Life (Letunic and Bork, 2019). Solid blue stars indicate presence of malignant cancers in the representative lineages. We coded the data to represent presence (filled in colored box) or absence (outline box) of the top reported human cancers, organized by tissue. GI, gastrointestinal.

**Figure 3:**
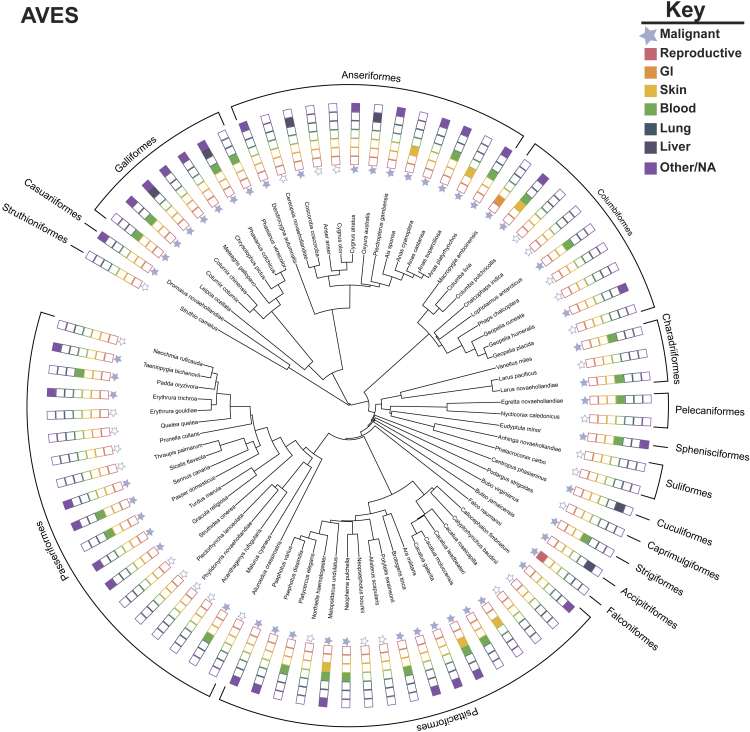
Phylogeny of Reported Cancers and Benign Tumors in Aves Illustration of the comprehensive Madsen et al. (2017) literature review of reported neoplasia/tumors and malignancy in birds. Species represented in the phylogeny had evidence of tumors. Phylogeny was inferred from VertLife (Jetz et al., 2012) and created using the Interactive Tree of Life (Letunic and Bork, 2019). Solid blue stars indicate presence of malignant cancers in the representative lineages. We coded the data to represent presence (filled in colored box) or absence (outline box) of the top reported human cancers, organized by tissue. GI, gastrointestinal.

**Figure 4:**
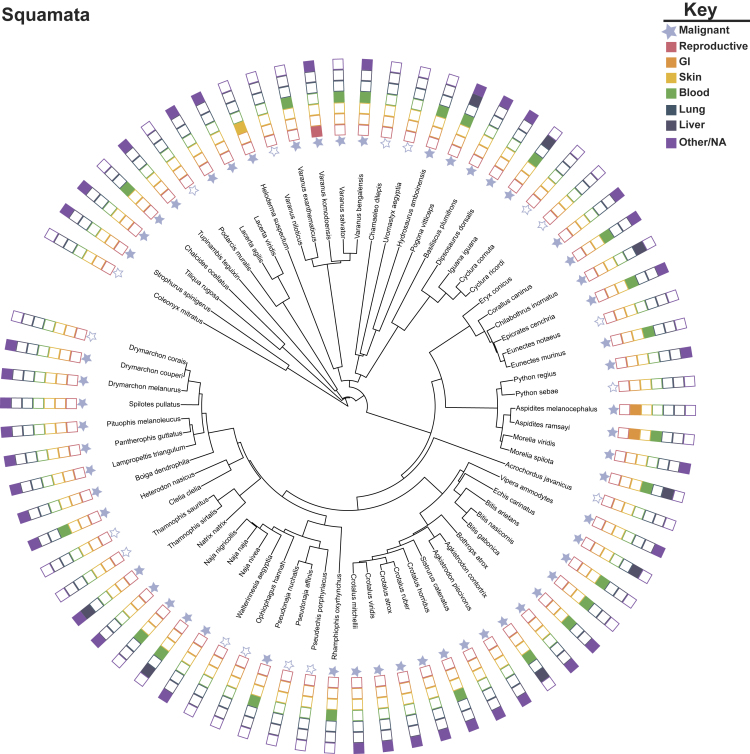
Phylogeny of Reported Cancers and Benign Tumors in Squamata Illustration of the comprehensive Madsen et al. (2017) literature review of reported neoplasia/tumors and malignancy in reptiles. Owing to limited data and phylogenies, we have only illustrated the Order Squamata (lizards and snakes) in this figure. Species represented in the phylogeny had tumors reported. Phylogeny was inferred from VertLife (Tonini et al., 2016) and created using the Interactive Tree of Life (Letunic and Bork, 2019). Solid blue stars indicate presence of malignant cancers in the representative lineages. We coded the data to represent presence (filled in colored box) or absence (outline box) of the top reported human cancers, organized by tissue. GI, gastrointestinal.

Analysis of data from previously published reviews on neoplasia and malignancy in managed and wild animals, suggests cancer is widespread across the animal kingdom (Aktipis et al., 2015; Albuquerque et al., 2018; Madsen et al., 2017). For example, we curated a dataset from the most recent literature review (Madsen et al., 2017) to focus on the top cancers diagnosed in human populations (Bray et al., 2018), including cancers of the reproductive tissues (ovary, testes, uterine, vulva), gastrointestinal tissues (GI), skin, blood, mammary tissues, lung, and liver (including bile duct). These data have limitations, including limited information on specific diagnoses and age/sex of the animal. Owing to these constrictions, we focused on the presence or absence of neoplasia (including benign tumors), along with malignant phenotype in Amniotes, including mammals (Figure 2), birds (Figure 3), and reptiles (Figure 4). Although there is still much work to do to survey cancer data in animals, including more quantitative analyses, we hope this summary provides a starting point and highlights general trends in the reviewed case reports.

Of the 223 species of mammals with reported neoplasia in this most recent literature review (Madsen et al., 2017), 165 of these species had evidence of malignancy (Figure 2). When we analyzed these data for cancer in all mammals, we found that the top tissues with reported malignancy included blood and liver/bile duct. Of particular interest, liver carcinomas were frequently reported in primates. Liver carcinoma was also previously reported as a common cancer diagnosis in a subset of primates, lemurs and lorises (Zadrozny et al., 2010). Although reports of mammary cancers were relatively infrequent in this dataset (compared with human data reported elsewhere), we highlight a cluster of mammary carcinomas in felid species, including tigers and lions. Mammary carcinoma in felids was attributed to the historical use of progesterone for contraception (McAloose et al., 2007). Owing to its association with mammary carcinomas, this contraceptive is no longer used. A substantial decrease in the prevalence of mammary carcinoma in felids occurred when progesterone was discontinued as a contraceptive (Harrenstien et al., 1996).

Of the 126 species of birds with reported neoplasia in the literature review (Madsen et al., 2017), malignancy was reported in 80 species (Figure 3). The most frequently diagnosed cancer in Aves was cancer of the blood. Skin was the second most common tissue with reported malignancy. Reproductive cancers were reported relatively rarely in birds compared with mammals, and there are no reports of lung cancer in the current review of bird cancer cases. These data were collected opportunistically, and we should be cautious to suggest Aves are protected from certain types of commonly reported cancers in humans, such as lung cancer. Instead, we would like to highlight these observations are worthy of follow-up investigation and validation.

Lastly, of the 138 species of reptiles with evidence of neoplasia in Madsen, 102 species had evidence of malignancy (Figure 4). Similar to birds, the most frequently reported cancer in reptiles was found in the blood. The second most frequently reported diagnosis was cancer of the liver and bile ducts. Interestingly, lung cancer was also not reported in the current cancer case study literature in reptiles. We should note that no reports of lung cancer in these animals does not mean they are protected from developing this type of cancer, because we do not know if all reptiles' lungs were thoroughly screened for cancer. Instead, these results should motivate more systematic data collection to verify malignancy rates in reptiles.

From the current, limited data on wildlife cancer, mammals appear to be more susceptible to cancer than other vertebrates (Aktipis et al., 2015; Albuquerque et al., 2018; Effron et al., 1977). As highlighted above, mammals may be more susceptible to reproductive cancers than either birds or reptiles. We recognize that this pattern could be due to sampling bias (as outlined in Hochberg and Noble, 2017), because mammals are charismatic animals. Mammals may be more likely to receive full and complete necropsies with histopathology compared with non-mammals. Currently, little empirical evidence exists to potentially explain why mammals may be more susceptible to cancer. However, multiple predictions have been proposed. Cancer could be cost to becoming a placental animal. Placentation and embryo implantation share similar biological processes to malignancy, including growth, invasion, and vascularization (D'Souza and Wagner, 2014; Haig, 2015; Kshitiz et al., 2019). These observations suggest mammals may be more susceptible to tissue-specific malignancy, such as tumors originating from epithelial origins; however, better comparative pathology data are needed to test this. Additionally, mammals have modified immune surveillance, because immune tolerance expands beyond self to prevent rejection of progeny during pregnancy. Mammals have a unique system to support maternal-fetal tolerance, including specific MHC molecules, such as HLA-G/HLA-C, which prevent fetus (non-self) rejection during pregnancy (Bubanovic and Najman, 2005). Some mechanisms of immunotolerance to tumors and fetuses are similar (Jørgensen et al., 2019), suggesting a link between tolerance to pregnancy and cancer vulnerabilities. Interestingly, a study found pregnancy transiently increases a women's risk for developing breast cancer (Nichols et al., 2019). It was recently proposed that a post-fertile lifespan, such as menopause, in mammals could arise as an adaption to defending against cancer in species with high parental investment (Thomas et al., 2019). Future work on the evolution of placentation, comparative biology of pregnancy and immune system in mammals could help explore the link between gestation and cancer susceptibility.

The applications of comparative oncology, especially characterizing tissue-specific cancer risk across species, could help answer fundamental questions in cancer biology and evolution. According to the “Bad Luck” hypothesis in cancer biology, a correlation exists between cancer risk per tissue and lifetime number of stem cell divisions within each tissue, suggesting cancer risk among tissue types can be explained by bad luck mutations (i.e., deleterious mutations accumulating during DNA replication) (Tomasetti et al., 2017; Tomasetti and Vogelstein, 2015). However, this hypothesis has been debated as a potential oversimplification, because various tissue types may differ in the accumulation of cancer-causing mutations (Noble et al., 2015). In addition, the Bad Luck hypothesis does not consider the effect of clonal expansions driven by oncogenic mutations, nor does it consider the fitness landscape of the somatic tissue (DeGregori, 2018; Rozhok et al., 2015). This stem cell theory focuses on human cancer, and little is known about the number of stem cell divisions in animal tissues. Future work to test the total number of stem cell divisions per anatomical site across species and its relationship to tissue-specific cancer risk would be an important future direction in comparative oncology. However, data on comparative tissue development, stem cell number, somatic mutation rates, and immunosurveillance in animals, along with highly curated cancer prevalence data, are needed to test the Bad Luck hypothesis across species.

## Evolution of Cancer Defenses across the Tree of Life

Comparative oncology offers a unique opportunity to observe and understand how evolution, over millions of years, resulted in differences in cancer defense mechanisms across species. Understanding human evolutionary shortcomings for cancer suppression mechanisms could potentially be leveraged to develop more effective, less toxic treatments for people with cancer. Most therapeutic approaches to cancer focus on treatment after diagnosis, which often occurs after spread of the primary tumor to other locations in the body. Metastatic cancer remains clinically challenging to treat and is often a threat to the survival of the patient (Mehlen and Puisieux, 2006). Rather than attempt to treat an evolving tumor, the ultimate goal of an evolutionary medicine approach is to leverage the lessons we learn from nature to prevent cancer from developing in humans and other animals. We highlight a few of the many promising lessons from nature below.

Humans are not devoid of our own cancer suppression mechanisms. Many of the mechanisms discovered in animals with the extraordinary ability to suppress cancer are also mechanisms found to varying degrees in humans. However, the activity of these cancer suppression mechanisms can often be enhanced in some animals compared with humans. For example, human cells express one of our most important tumor suppressor genes, *TP53* (Kastenhuber and Lowe, 2017; Lane and Levine, 2010). Functional activity of this gene is lost in more than half of all human tumors (Petitjean et al., 2007). A potential mechanism of cancer resistance in large and long-lived elephants is the evolution of extra copies of this critical tumor suppressor, in the form of retrogenes. One of the essential functions of *TP53* is to induce apoptosis of cells with damaged DNA. Elephant (*Loxodonta africana* and *Elephas maximus*) cells with DNA damage undergo more apoptosis than human cells, in part due to enhanced *TP53* activity (Abegglen et al., 2015). Increased sensitivity to DNA damage likely protects elephants from cancer by preventing the accumulation of cancer-causing mutations.

Approaches to increase *TP53* function in human tumors have been pursued over the last few decades with promising treatments in clinical trials (Cheok and Lane, 2017; Hientz et al., 2017; Khoo et al., 2014). Mice with an extra truncated but active copy of *Trp53* (mouse version of *TP53*) provide support that enhanced *TP53* activity suppresses cancer. When the extra copy of *Trp53* was expressed under a constitutive promoter (continuous expression), a trade-off was observed. Although these mice developed cancer at a lower rate than mice with the normal two copies of *Trp53*, they aged prematurely, possibly due to excessive apoptosis induced by *Trp53* (Tyner et al., 2002). However, when an extra copy was expressed under the *Trp53* promoter (not continuous expression), the mice still developed less cancer, but the premature aging phenotype was not observed (García-Cao et al., 2002, p. 53). These results stress the importance of proper regulation of *TP53* expression and function. Understanding *TP53* retrogene expression and function and the mechanism of enhanced *TP53* activity in elephants could reveal unique therapeutic approaches to increase *TP53* function in human cancer cells and perhaps even in normal cells to kill damaged cells before cancer develops.

Long-lived bats are another promising species for comparative oncology research. Many bat species have extraordinary life history traits, such as an extended lifespan for an animal of its body size (Wilkinson and Adams, 2019). For example, the maximum lifespans of bats are at least three times longer than that of nonflying eutherians (placental mammals) (Austad and Fischer, 1991). Based on their extraordinary longevity, it has been suggested that bats develop cancer at very low rates (Tollis et al., 2017). Recently, it was discovered that bat cells do not accumulate DNA damage in response to chemotherapeutic drugs (etoposide and doxorubicin). This unusual response to chemotherapeutic drugs may be due to increased expression of an ABC transporter, ABCB1, which is a cell membrane protein that traffics foreign substances out of cells (Hodges et al., 2011). This mechanism, which is conserved across multiple species of bats, may protect them from developing cancer by preventing DNA damage that could accumulate with exposure to toxins and carcinogens, such as pesticides ingested when feeding on fruit or insects. Human cells also express the ABCB1 protein, and interestingly, tumors in humans often increase expression of this gene as a mechanism to resist chemotherapeutic treatment (Wijdeven et al., 2016). It is ironic that a mechanism that potentially protects bats from developing cancer is also the same mechanism used by tumor cells to become more aggressive and resistant to treatment. Although the efficacy of chemotherapeutic drugs depends on low activity of ABC transporters, this mechanism could be employed to protect normal cells from the harmful DNA damaging effects of these genotoxic cancer drugs. Temporarily increasing ABCB1 expression in normal cells but not tumor cells during chemotherapeutic treatment, if possible, could be a chemoprotective approach in patients with cancer. This chemoprotective approach could decrease the incidence of treatment-induced, secondary cancers that can develop in patients with cancer years after successful treatment owing to systemic exposure to DNA damaging chemotherapeutic drugs (Vega-Stromberg, 2003).

Naked mole rats (*Heterocephalus glaber*) are one of the most well-known (nearly) cancer-resistant species in the animal kingdom. Decades of research on these eusocial mammals have only recorded a few rare instances of neoplasia (Buffenstein, 2005; Delaney et al., 2016). Researchers have uncovered multiple potential mechanisms that naked mole rats may have evolved to suppress cancer. One of these mechanisms involves the expression of a component of the extracellular matrix, hyaluronic acid (HA). Although human cells also secrete HA into the extracellular matrix, naked mole rat (NMR) cells secrete a very-high-molecular-weight version of HA (vHMW-HA). vHMW-HA protects NMRs from cancer by making their cells hypersensitive to contact inhibition (Tian et al., 2013). The assembly of HA in NMRs shows elastic, supercoiled structures not found in other animals and likely contributes to increased cell contact inhibition. The recent characterization and understanding of NMR HA structure could enable the synthetic engineering of biopolymers to mimic this structure. If we can successfully replicate the NMR HA structure in human tissues with little to no toxicity, then we can potentially develop a powerful cancer therapeutic to prevent cellular invasion and metastasis (Kulaberoglu et al., 2019), which are responsible for most cancer-related deaths (Welch and Hurst, 2019).

In addition to the mammals highlighted above, another extraordinary animal with incredible resistance to DNA damage is the nearly indestructible, microscopic tardigrade. Tardigrades have multiple protective mechanisms, such as desiccation and temperature tolerance (Boothby, 2019), that allow them to survive in extreme, radioactive environments (Jönsson, 2019). Interestingly, one particular mechanism tardigrades use to protect their DNA from environmental damage is absent in humans. Tardigrades express a unique protein called Dsup (for damage suppressor) (Hashimoto et al., 2016) that binds to nucleosomes and prevents DNA damage in tardigrade cells by protecting chromatin from hydroxyl radical-mediated DNA cleavage (Chavez et al., 2019). The lack of sequence similarity to any known proteins or motifs suggests that Dsup is a protein that evolved only in tardigrades (Hashimoto et al., 2016). Expression of Dsup in human embryonic kidney (HEK293) cells significantly suppressed DNA damage induced by radiation compared with cells that did not express Dsup (Hashimoto et al., 2016). These results suggest that mimicking the activity of this protein in human cells, if possible, could protect DNA from radiation-induced damage and reduce the incidence of radiation-induced cancers.

Crocodiles and alligators are additional species rarely observed to develop cancer. Although there is limited evidence of cancer incidence in crocodilians, recent models suggest they are excellent candidates for comparative oncology research (Brown et al., 2015). For example, crocodiles and alligators evolved extraordinary life history traits, and unlike most animals across the tree of life, their probability of dying *decreases* with age (Briggs-Gonzalez et al., 2017), despite living in unsanitary, sometimes contaminated environments (Siddiqui et al., 2017). Serum from crocodilians was discovered to possess anti-microbial and anti-tumor properties (Siddiqui et al., 2017). Recently, attempts to identify the serum compound responsible for these effects revealed that white blood cells extracts from crocodiles contain two unique anti-microbial peptides, Leucrocin I and II (Pata et al., 2011). Leucrocin I was modified to improve its anti-microbial activity. There is evidence that the modified peptide, RT2, kills human cancer cells *in vitro* and *in vivo* (Maijaroen et al., 2018; Maraming et al., 2018). Although we should cautiously interpret these preliminary studies, which need replication and validation with a negative control peptide, naturally evolved mechanisms of cancer resistance in crocodilians may still hold great promise. Many additional mechanisms of cancer suppression across species await discovery. Using a comparative approach, we can begin to identify which known parameters (i.e., DNA damage response or protection from DNA damage) are potentially more exploitable for human cancer prevention and treatment (Somarelli et al., 2020a, 2020b; Stenvinkel et al., 2018).

## Shared Planet, Shared Health: Cancer Treatment in Non-human Patients

Translational work from comparative oncology extends beyond attempts to improve human patient outcomes. It is also important for improving animal patient healthcare, as well as species conservation (Garden et al., 2018). Currently, zoological and aquarium institutions routinely perform preventive medical examinations to diagnose and treat animals with cancer. When an animal is diagnosed with cancer, veterinarians collaborate with veterinary oncologists to develop a treatment plan. Similar to human patients, researchers are beginning to sequence DNA from non-human tumors to understand what alterations are shared, as well as unique, in tumor genomes between species (Gardner et al., 2019; Rao et al., 2020). Understanding tumor driver mutations will help veterinary oncologists tailor treatments to the individual animal, similar to targeted therapies used to treat humans with cancer. In addition to better, more personalized treatment of animal patients, there is an opportunity to leverage animal clinical trial data as better models (compared with traditional human preclinical models, including mice) to improve clinical trial results and increase new drug approvals to help treat both human and animal patients (as reviewed in Somarelli et al., 2020a, 2020b).

To improve our understanding of comparative oncogenesis, collaboration and data sharing between veterinarians, researchers, cancer biologist, wildlife biologists, evolutionary biologists, genomicists, as well as clinicians, is required (Somarelli et al., 2020a, 2020b). Standardization of medical data collection across human and animals, including diagnoses and terminology, is desperately needed to improve the efficiency and feasibility of comparative studies. Currently, a centralized database (www.escra.org) is available for participating zoos and institutions to document standardized comparative oncology data and to help veterinary oncologists find the best treatment options. However, not all zoos and institutions use this database, and complete standardization of medical data collection will require universal buy in. Working together to understand cancer in human and animal patients can bridge the gaps in the field of comparative oncology.

## Conclusion

Comparative oncology is a promising field of cancer biology. Using tools from evolutionary theory, we are beginning to understand what makes certain species more vulnerable to neoplasia than others. Species with extraordinary life history traits, such as extended lifespan or large body mass, are excellent candidates for future investigations to characterize novel and shared cancer defense mechanisms. From current studies, we report that neoplasia and malignancy are widespread across mammals, birds, and reptiles, with blood cancers reported most frequently. We highlight a few examples of recently discovered cancer suppression mechanisms in animals, such as heighten sensitivity to DNA damage and novel DNA damage suppression proteins and the potential for translation into effective treatments. Although challenges associated with obtaining cancer data in both zoological and wild animals exist, we must overcome these challenges. Many additional mechanisms of cancer suppression across species remain to be discovered. These discoveries have tremendous potential to improve outcomes for all animals with cancer, including humans. Collaborations between animal wildlife experts, zoological institutions, ecologists, veterinarians, oncologist, evolutionary biologists, and other researchers will be the key in facilitating progress in the promising field of comparative oncology.

## Methods

All methods can be found in the accompanying Transparent Methods supplemental file.
